# Performance of the new SmartCardia wireless, wearable oximeter: a comparison with arterial SaO2 in healthy volunteers

**DOI:** 10.1186/s12871-022-01604-w

**Published:** 2022-03-24

**Authors:** Francisco Rincon, Julien Pidoux, Srini Murali, Jean-Jacques Goy

**Affiliations:** 1 Cardiology Department of Clinic Cecil, Av. Ruchonnet 53, 1003 Lausanne, Switzerland; 2 EPFL, Innovation Center, Lausanne, Switzerland; 3 University Hospital, Fribourg, Switzerland

**Keywords:** Oximetry, Nellcor device, Masimo device, SpO_2_ measurement, SmartCardia

## Abstract

**Background:**

Our goal was to evaluate the performance of a new wearable arm located pulse oximeter.

**Methods:**

Twelve volunteers were monitored with three pulse oximeters and underwent desaturation to 70% SaO_2_.

We compared the accuracy of SpO_2_ reading from the SmartCardia system with SpO_2_ using two well established devices (Masimo and Nellcor) as reference.

Oximetry was performed at different level of oxygen saturation varying from 70 to 100%. Bias, ARMS and precision were evaluated using Bland-Altman plots.

**Results:**

The mean (SD) differences between SaO_2_ compared to SpO_2_ and the devices were as follows: SaO_2_ versus Masimo 2,12 ± 1,01% (95% CI 1,45 to 2,79), SaO_2_ versus Nellcor 0,78 ± 0,58% (95% CI − 0,29 to 1,65) and SaO_2_ versus SmartCardia 0,42 ± 0,24% (95% CI − 0,64 to 1,46). The bias between SmartCardia, Masimo, Nellcor devices and SaO_2_ was 0.16 (95% CI 0.05 to 0.33) and LoA (level of agreements) 2.96 (95% CI − 2,68 to 2,89) for SmartCardia, 2,02 (95% CI 1,49 to 2,54) and LoA − 6 to 11 for Masimo, and 0,76 (95% CI 0,5 to − 1) and LoA − 3,5 to 5,0 for Nellcor. ARMS for the 70–100% SaO_2_ range was 1,4 for SmartCardia, 5,0 for Masimo and 2,31 for Nellcor.

**Conclusions:**

The new wireless SmartCardia SpO_2_ measurement system demonstrated in-line results, bias, ARMS and precision in healthy volunteers, when compared with the gold standard SaO2 and with two well established systems, Masimo and Nellcor.

**Trial registration:**

The present trial was prospectively registered at UCSF record (registration number:10–00437), on March 8, 2021.

## Introduction

Pulse oximetry is a procedure for measuring the level of oxygen in the blood expressed as oxygen saturation [1–3]. This parameter is widely used in the evaluation of various medical conditions that affect the function of the heart and lungs and is a standard monitoring modality during anesthesia delivery [4–6].

Conventional oximeters use either the reflective or the transmitive method to measure SpO2. The reflective method is known to be less accurate. For this reason, most commercially available patches do not provide SpO2 measurements in addition to cardiac monitoring. The transmissive method is the more commonly used of the two. Transmitive technology transmits red and infrared light through the finger to a photo detector. The pulse oximeter sensor reads the transmitted light beams to estimate oxygen saturation of the blood and pulse rate. This method positions the transmitter and receiver in the same plane and the sensors can be placed on areas of the anatomy other than the finger. Conventional pulse oximeters have various limitations including false alarm and failed measurements [7–11] which may be related to positioning of the device located on the finger. A new generation of reflective pulse oximetry device (SmartCardia oximeter) has been designed on a wireless basis [12, 13]. The present trial was designed to validate this technology and to demonstrate the potential of this device using the reflective method. We compared this new device to the Nellcor N-600 (Covidien, Boulder, CO, USA) and the Masimo Radical device (Masimo Corp., Irvine, CA, USA), both units considered the two most used and accurate FDA approved non-invasive devices for SpO_2_ measurements. SaO2 was used as the gold standard.

## Methods

### Ethical considerations

Even if the device was developed in Switzerland the trial was conducted in a reference laboratory accepted by the FDA, the Hypoxia Research Laboratory at UCSF. The study was reviewed and approved by the UCSF Ethical Committee on Human Research. The Approval number is 10–00437, expiring on March 8th, 2021. The approval letter is on file at UCSF. The laboratory conforms to Good Clinical Practice Standards for the involvement of human subjects and handling of test data. Written informed consent was obtained from each participant.

### System description

**Fig. 1:**
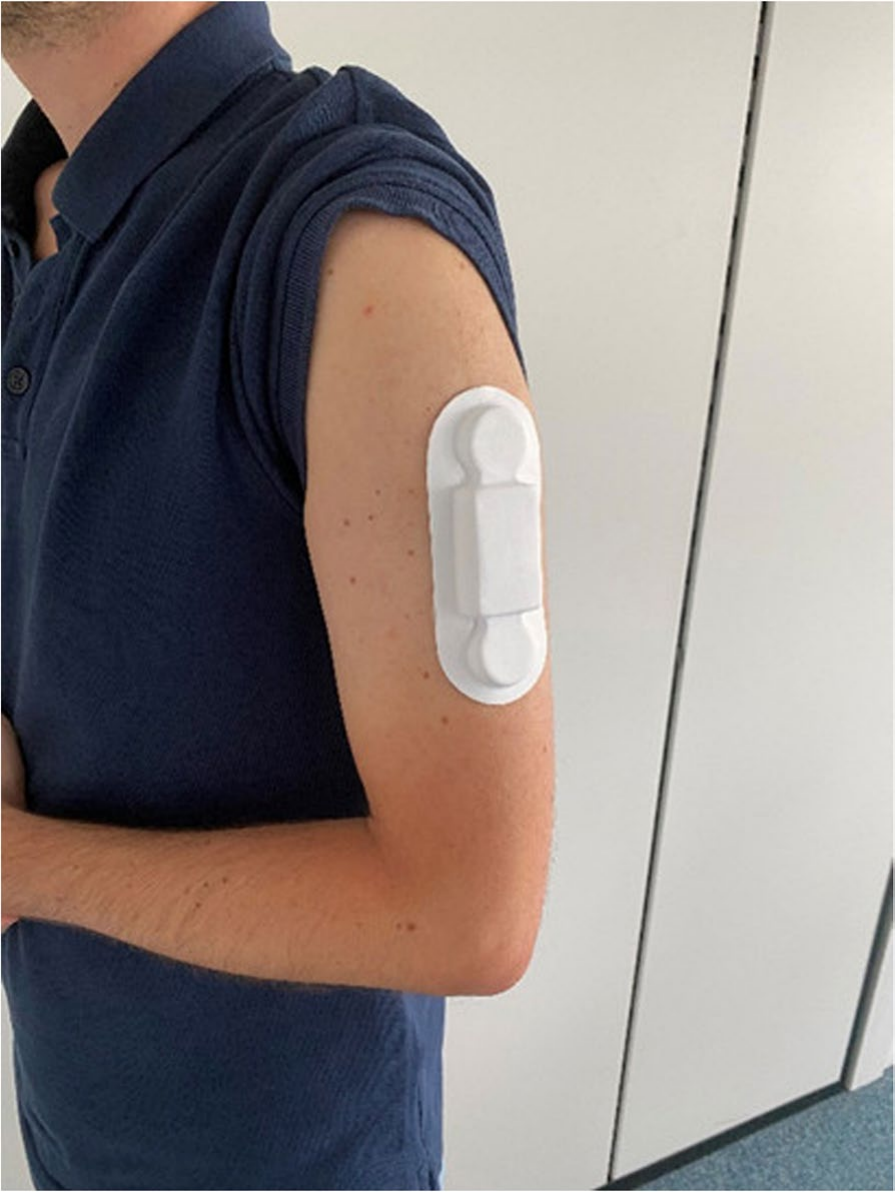
SmartCardia device especially dedicated for SpO2 measurement

The SmartCardia 7 L device is a wireless patch with a low-cost disposable component and a re-usable electronic unit (Fig. 1). The patch acquires ECG and measures heart rate (HR) and SpO_2_. The data are transmitted by Bluetooth to a mobile phone or router. The measured signals and parameters are also stored on the device. The device for SpO_2_ recording is placed on the left arm of the subject (Fig. 1). The size of the patch is 55 × 130 mm. The patch-based device offers up to 14-days monitoring and data storage and 7-days real-time connectivity with the cloud through a smartphone. The ability to receive, store, and interpret a broad range of signals offers the opportunity to go far beyond monitoring individual parameters. If the patient’s vital measurements reach a pathological value, the system gives an alert on the cloud and the physician can see the real-time parameters and ECG signals.

### Protocol

The protocol required brief stable arterial oxygen desaturation in healthy volunteers and sampling arterial blood when a stable level of hypoxia has been attained. The blood sample was analyzed for oxygen saturation with a gold standard bench CO-oximeter, currently a Radiometer ABL-90 multi-wavelength oximeter (Hemoximeter, Radiometer, Copenhagen (Denmark), serial 1393-090R0359N0002). This instrument contains factory certified calibration standards and quality control algorithms. Twenty to twenty-five arterial blood samples from each subject can be analyzed following a protocol aligned with current ISO and FDA guidance documents for pulse oximeter testing.

A radial arterial cannula was placed in either the left or right wrist of each subject for arterial blood sampling and blood pressure monitoring.

Our approach to obtaining stable, safe, and controlled hypoxia was breath-by-breath respiratory gas analysis. A computer program permits the inspired gas mixture to be adjusted to achieve a level of lung alveolar gas that will achieve the desired degree of hypoxia. This was obtained with the use of a nonerebreathing circuit with CO2 removal. Typically, saturation is determined once with air breathing and then at each of the following levels, e.g., 93, 90, 87, 85, 82, 80, 77, 75 and 70% saturation for about 30–60 s at each level. An arterial blood sample is obtained from the catheter at the end of each hypoxic plateau. The operator changes the inspired oxygen concentration at the end of each blood sampling to attain the next desired steady-state hypoxic condition. A run takes 10–15 min, and each run is terminated by a breath of 100% O_2_ followed by room air. Two runs together enable obtaining a total of 20–25 blood samples, two samples at each different plateau. Saturation of each arterial blood sample is determined by direct oximetry using the Radiometer ABL-90 multi- wavelength oximeter.

Sites for affixing pulse oximeter probes was the fingers for the Masimo device, the ears for the Nellcor, and the arm for the SmartCardia. In order to avoid inaccurate readings due to head or fingers movements with these 2 devices, the arm of the subjects was fixed during the measurements period. In addition, the participants were asked not to move their heads, as much as possible for the duration of the measurements.

### Statistics

Pulse oximeter data was taken as 5 s averages corresponding to the point of SaO_2_ analysis. Individual data points may be missed or excluded for dropped signals or failure of the oximeter signal to achieve an appropriate plateau. Agreement in SpO_2_ and arterial SaO2 were analyzed using Bland Altman analysis [14, 15]. Bland Altman curve gives a graphical representation of the agreement between SpO_2_ and arterial SaO2 value. The average of the SpO_2_ and SaO_2_ is plotted on the x-axis while the difference between the two values is plotted on the y-axis. The more the graph points toward a zero difference with narrow dispersion of the ‘body’, the better the agreement.

Tables of mean, standard deviation, standard error, minimum, maximum, 95% CI, count and root mean square are provided for each oximeter’s bias, and all oximeters combined in the following ranges of SaO2: 70–100%, 60–70%, 70–80%, 80–90%, and 90–100%. LoA was obtained between methods of measurement with multiple observations per individual [16]. Root mean square error (ARMS) was calculated as it represents the best way to compare SaO2 measurements. It includes both values of SaO2 and its stability. The following formula was used for calculation:

$$\mathrm{ARMS}=\sqrt{\frac{\sum {\left({\mathrm{SpO}}_2-{\mathrm{SpO}}_2\right)}^2}{\mathrm{n}}}$$

Data were managed on MS excel spreadsheet and analysed using stata 9.0 software (Stata Corp, College Station, TX). On Bland Altman curves linear regression is shown for all subjects combined and the equation with R2 is shown on the plot. Mean bias is displayed as a solid horizontal line, and the upper and lower limits of agreement (mean bias ±1.96•SD*) are shown by dashed horizontal lines. For “pooled” plots, different markers are used for each pulse oximeter. Continuous variables were compared using ‘t’ test. A *p* value < 0.05 was considered as significant.

## Results

The current study was performed on 4/13/2021 and 4/14/2021 at UCSF. Twelve volunteers with a mean age of 28 (range 21–29) (3 women and 9 men) were included in the trial (Table 1). All subjects enrolled in the study had normal Hb level (Hb ≥ 10 g/l) and all were healthy and non-smoking individuals.

**Table 1:** Mean ± SD SpO2 values at different plateaus with the 3 oximeters for the 12 subjects. Mean differences between the 3 oximeters are shown as delta

Oxymeter	H SaO2 in %	SC SpO2 in %	Ma SpO2 in %	Ne SpO2 in %	Delta H/SC	Delta H/Ma	Delta H/Ne
Baseline	97,83 ± 0,43	97,85 ± 0,85	98 ± 2,92	97,9 ± 1,2	0,02	0,17	0,07
Plateau 1	94,81 ± 1,33	94,58 ± 1,39	96,75 ± 2,38	94,83 ± 1,45	−0,23	1,94	0,02
Plateau 2	91,25 ± 2,48	90,78 ± 3,45	93,75 ± 3,11	91,54 ± 3,02	−0,47	3,5	0,29
Plateau 3	87,14 ± 3,04	87,09 ± 3,32	89,29 ± 3,99	87,25 ± 3,84	−0,05	2,15	0,11
Plateau 4	82,96 ± 3,25	83,16 ± 3,43	86,04 ± 4,62	84 ± 4,75	0,20	3,08	1,04
Plateau 5	76,90 ± 3,5	77,49 ± 3,67	79,5 ± 5,95	78,5 ± 4,93	0,59	2,6	1,6
Plateau 6	72,22 ± 3,42	73,03 ± 3,56	74,64 ± 5,86	72,79 ± 4	0,81	2,42	0,63
Break	99,28 ± 0,31	98,75 ± 0,59	98,791,28	98,75 ± 1,23	−0,53	−0,49	−0,53
Plateau 7	92,73 ± 2,81	92,49 ± 2,53	95,08 ± 2,84	93,15 ± 2,29	−0,24	2,35	0,42
Plateau 8	87,65 ± 2,28	87,81 ± 2,36	90,5 ± 3,32	88,42 ± 2,02	0,16	2,85	-0,23
Plateau 9	82,43 ± 2,77	82,82 ± 3,2	85,29 ± 5,09	84,05 ± 2,44	0,39	2,86	1,62
Plateau 10	76,26 ± 2,36	76,72 ± 2,21	78,71 ± 6,1	77,95 ± 2,33	0,46	2,45	1,69
Plateau 11	71,71 ± 2,59	73,06 ± 2,16	73,29 ± 7,92	73,45 ± 3,86	1,35	1,58	1,84
Mean					0,42 ± 0,24	2,12 ± 1,01	0,78 ± 0,58

*H* hemoximeter, *Ma* the Masimo system, *Ne* the Nellcor system, *SC* the SmartCardia system. Delta is the mean difference between SaO2 and the tested system

**Fig. 2:**
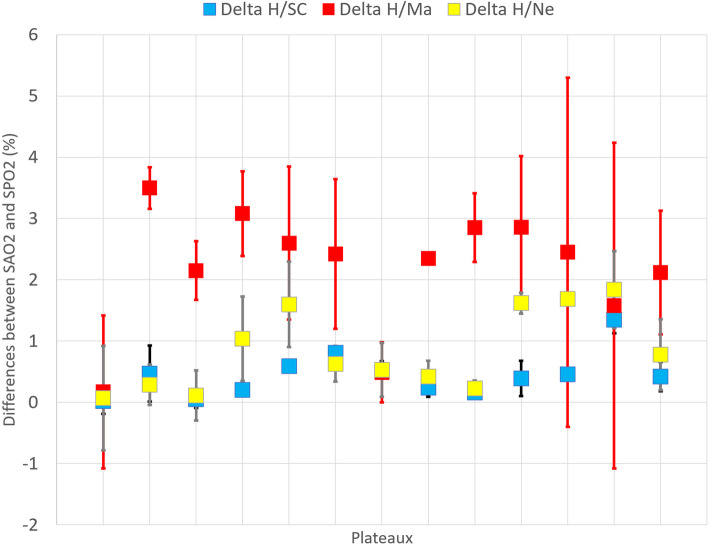
Plots showing agreement between SaO2 (H) and SpO2 detected by the SmartCardia device (SC), the Masimo device (Ma) and the Nellcor (N). Results +/− SD are presented as the difference between the device and the gold standard

A total of 298 samples were obtained at the saturation plateaus during the study. Data were collected for each type of oximeter and probes. Mean (SD) differences measurements between SaO_2_ and SpO_2_ and between the devices tested were as follow: SaO_2_ versus Masimo 2,12 ± 1,01% (95% confidence interval CI, 1,45 – 2,79), SaO2 versus Nellcor 0,78 ± 0,58% (CI, − 0,29 - 1,65) and SaO2 versus SmartCardia 0,42 ± 0,24% (95% CI, − 0,64 - 1,46). A statistically significant difference in precision was observed between the SmartCardia and the Masimo device (*p* < 0.001), between the SmartCardia and the Nellcor device (*p* = 0.03) and between the Nellcor and the Masimo (*p* < 0.001) (Table 1 and Fig. 2).

**Fig. 3:**
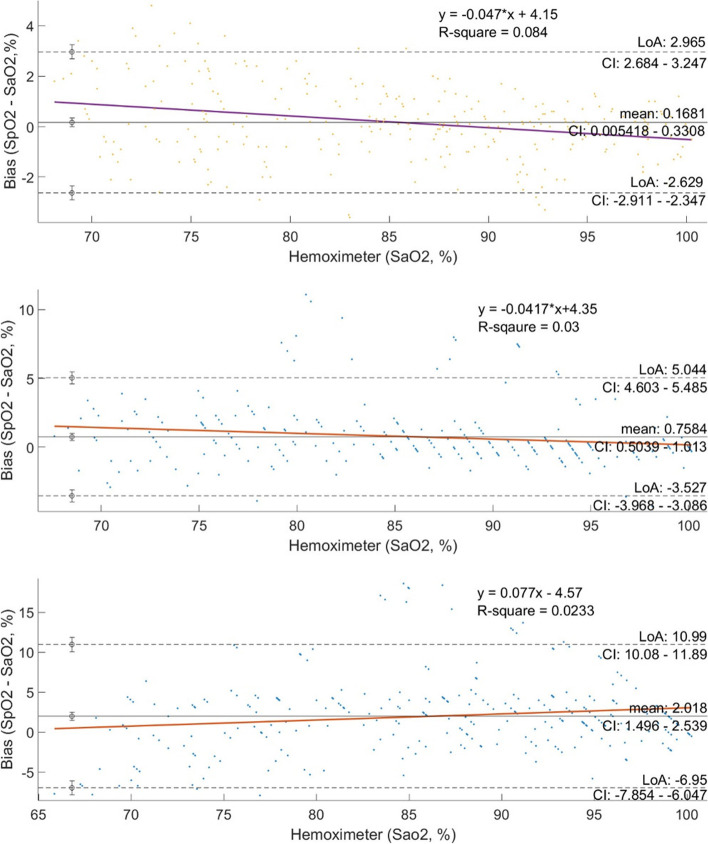
Bland-Altman curve showing agreement between **a**: SmartCardia, **b**: Masimo and **c**: Nellcor, SpO2 and arterial SpO2. Data is plotted as Hemoximeter data (SaO2) vs. pulse oximeter bias (SpO2 – SaO2). A different marker is used for each study subject. Linear regression is shown for all subjects combined, and the equation with R2 is shown on the plot. Mean bias is displayed as a solid horizontal line, and the upper and lower LoA (mean bias ±1.96•SD*) are shown by dashed horizontal lines

Bias, precision and ARMS are presented in Table 2. Plots assessing the agreement between arterial SaO_2_ and SpO_2_ detected by the SmartCardia, the Masimo and the Nellcor pulse oximeters are shown in Fig. 2. The bias between SmartCardia, Masimo, Nellcor and SaO_2_ was as follows: 0,16 (95% CI 0.05 to 0,33) and the LoA were 2,96 (95% CI − 2,68 to 2,89) for SmartCardia, 2,02 (95% CI 1,49 to 2,54) and the LoA were − 6 to 11 for Masimo, 0,76 (95% CI 0,5 to − 1) and the LoA were − 3,5 to 5,0 for Nellcor. This bias is significantly lower for SmartCardia compared to Masimo (*p* < 0.001) and Nellcor (*p* < 0.05), and for Nellcor compared with Masimo (*p* < 0.05). ARMS for the 70–100% SaO_2_ range was 1,4 for the SmartCardia device, 5,0 for the Masimo device and 2,31 for the Nellcor device (Table 2 and Fig. 3).

**Table 2:** Bias and ARMS at different oximetry ranges with the 3 oximeters compared with SaO2

Hemoximeter-Range (%)	60–70	70–80	80–90	90–100	70–100
**SaO2 versus SmartCardia**					
Mean ± Standard deviation Range: (Minimum, Maximum)	1.82 ± 0.94 (0.3;3.4)	0.57 ± 1.81 (−2.9; 4.8)	0.17 ± 1.23 (−3.6;3.1)	−0.32 ± 1 (−3.3;1.5)	0.17 ± 1.4 (− 3.6;4.8)
Count	12	86	91	109	286
Missing Data	0	0	1	1	2
Root Mean Square	2.03	1.88	1.23	1.04	1.4
**SaO2 versus Masimo**					
Mean ± Standard deviation Range: (Minimum, Maximum)	1.93 ± 3.6 (−7.7;6.4)	2.27 ± 6.55 (−7.8;18.6)	2.68 ± 4.19 (−7.9;13.7)	1.35 ± 2.63 (−5.8;9.5)	2.02 ± 4.6 (− 7.9;18.6)
Count	12	86	91	109	286
Missing Data	0	0	0	2	2
Root Mean Square	2.96	4.9	6.9	3.95	5.00
**SaO2 versus Nellcor**					
Mean ± Standard deviation Range: (Minimum, Maximum)	1.51 ± 1.52 (−1.7;3.9)	1.43 ± 2.94 (−3.9;11.1)	1.0 ± 2.1 (−2.0;8.0)	0 ± 1.3 (−4.3;5.5)	0.76 ± 2.19 (−4.3;11.1)
Count	12	86	91	109	286
Missing Data	0	9	3	2	14
Root Mean Square	1.29	2.32	3.26	2.1	2.31

*SD* standard deviation, *CI* confidence interval

## Discussion

Currently, arterial oxygenation is primarily measured using pulse oximeters that provide immediate and continuous non-invasive surveillance for SpO2. Several devices are available in clinics with some limitations, including false alarms and failed measurements when used in patients with low perfusion or during patient motion. The positioning of the fingertips makes measurements at times difficult and impractical for patients. Some trials showed varying sensitivity and accuracy in patients with hypotension and hypoxemia [14, 16], primarily due to the location of the device. We compared the performance of two well-established pulse oximeters to a next-generation pulse oximeter, the SmartCardia device. This wireless system is located on the patient’s arm and the values are transmitted electronically and stored in the cloud, both representing major benefits over other devices. Furthermore, today, as a result of the COVID-19 pandemic, the measurement of SpO2, even outside the hospital, has become increasingly important. We have shown that despite the use of the usually less accurate reflective method, the SmartCardia device provides very precise and precise results when compared to well-established devices in clinical use. Limitations due to the presence of wired connections are avoided, and patient hand movement is not limited by the presence of a finger mounted sensor. Furthermore, the oximeter can be worn during walking with permanent transmission of data to a control station or a phone providing reliable electronic wifi transmission. This allows access to permanent and uninterrupted information regarding patient oxygenation levels. To confirm the quality of the device, which is currently awaiting FDA approval, its performance was compared with the Masimo and Nellcor pulse oximeter devices. Both use the transductive method. In our study, the results are encouraging and equivalent or even slightly better based on bias, ARMS and precision measurements. We also showed that this system provides good SpO_2_ assessments over the range of SaO_2_ tested between 70 to 100% oxygen saturation.

Our trial involved young healthy volunteer subjects in a reference laboratory setting. This allowed the use of a more rigorous protocol while maintaining control over variables.

As mentioned before, this system is placed on the subject’s arm which is an advantage due to fewer disturbances related to the hand’s movements. Devices placed on the ear are also more prone to disturbances related to movements of the patient’s head. We found significant performance differences among the 3 devices tested. We showed that the Masimo device tended towards 2 to 3% higher SpO2 values than actual SaO_2_. This may be clinically relevant if confirmed in ICU patients. As our trial was not performed in a clinical setting, we cannot conclude that one oximeter would be better than the others in daily practice. Further trials in real life situations, such as ICU environments, hypotensive or hypothermic conditions, etc. are needed to further draw definitive conclusions. It is our experience that even well simulated clinical scenarios often lead to ambiguous results. We observed a significant difference in the measurement of SpO2 between the Nellcor and the Masimo in favor of the Nellcor system. However, our data were collected in healthy volunteers. Furthermore, previous trials comparing the Masimo, the Nellcor and the Philips Intellivue have not shown significant differences between these three pulse oximeters when tested in ICU patients [16, 17]. Conversely, other trials conclude to a higher sensitivity and specificity of the Masimo device when compared to the Nellcor system [18]. These divergent results clearly show the need for further studies utilizing real life environments and patient’s conditions to further corroborate our results.

### Study limitation

The major limitation is that we performed this trial in a laboratory. This may not fully replicate the characteristics of complex clinical settings exhibited by patient populations in the ICU or operating theater. In addition, the trial was conducted with a small number of patients. These results need confirmation in a larger cohort of subjects in a clinical setting.

## Conclusion

The new wireless SmartCardia SpO_2_ measurement system demonstrated in-line results, bias, ARMS and precision in healthy volunteers, when compared with the gold standard SaO2 and with two well established systems, Masimo and Nellcor.

## Data Availability

Data from the trail are stored on the UCSF files and can be consulted at any time. The datasets used and/or analysed during the current study available from the corresponding author or from Francisco Rincon on reasonable request.’

## Abbreviations

Hb: Hemoglobin blood level
UCSF: University of California, San Francisco
CI: Confidence interval
ICU: Intensive care unit
SpO2: Saturation measured by the tested oximetry device
SaO2: Saturation measured by the Hemoximeter
LoA: Limits of agreements
ARMS: Root mean square error

## References

[1] Jubran A (2004). Pulse oximetry. Intensive Care Med.

[2] Neff TA (1988). Routine oximetry. A fifth vital sign?. Chest.

[3] Chan ED, Chan MM, Chan MM (2013). Pulse oximetry: understanding its basic principles facilitates appreciation of its limitations. Respir Med.

[4] Eichhorn JH, Cooper JB, Cullen DJ (1986). Standards for patient monitoring during anesthesia at Harvard Medical School. JAMA.

[5] Runciman W (1993). International Task Force on Anaesthesia Safety. Eur J Anaesthesiol Suppl.

[6] World Federation of Societies of Anaesthesiologists (2008). International Standard for a Safe Practice of Anaesthesia.

[7] Lawson D, Norley I, Korbon G, Loeb R, Ellis J (1987). Blood flow limits and pulse oximeter signal detection. Anesthesiology.

[8] Severinghaus JW, Spellman MJ (1990). Pulse oximeter failure thresholds in hypotension and vasoconstriction. Anesthesiology.

[9] Wilson S (1990). Conscious sedation and pulse oximetry: false alarms?. Pediatr Dent.

[10] Plummer JL, Zakaria AZ, Ilsley AH, Fronsko RR, Owen H (1995). Evaluation of the influence of movement on saturation readings from pulse oximeters. Anaesthesia.

[11] Wiklund L, Hök B, Ståhl K, Jordeby-Jönsson A (1994). Post anesthesia monitoring revisited: incidence of true and false alarms from different monitoring devices. J Clin Anesth.

[12] Murali S, Rincon F, Cassina T, Cook S, Goy JJ (2020). Heart Rate and Oxygen Saturation Monitoring with a New Wearable Wireless Device in the Intensive Care Unit: Pilot Comparison Trial. JMIR Biomed Eng.

[13] Murali S, Brugger N, Rincon F, Mashru M, Cook S, Goy JJ (2020). Cardiac ambulatory monitoring: new wireless device validated against conventional Holter monitoring in a case series. Front Cardiovasc Med.

[14] Bland JM, Altman DG (1999). Measuring agreement in method comparison studi*es*. Stat Methods Med Res.

[15] Bland JM, Altman DG (2007). Agreement between methods of measurement with multiple observations per individual. J Biopharm Stat.

[16] Ahrens T, Ott K (2006). Comparison of three new generation pulse oximeters in a medical intensive care unit. Crit. Care Med.

[17] Shah N, Ragaswamy H, Govindugari K, Estanol L (2012). Performance of the three new-generation pulse oximeters during motion and low perfusion in volunteers. J Clin Anesth.

[18] Bipin J, Rakesh L, Kabra S (2014). Comparison of two new generation pulse oximeters with arterial oxygen saturation in critically ill children. Indian J Pediatr.

